# Supplementary material for: Bayesian Nonparametric Sensitivity Analysis of Multiple Test Procedures Under Dependence
Source: Biom J. 2025 Dec 14;67(6):e70101. doi: 10.1002/bimj.70101 (PMC12703229; doi:10.1002/bimj.70101)
Supplement: Supplementary file 1 — Supporting File: bimj70101‐sup‐0001‐DataCode.zip [file BIMJ-67-e70101-s001.zip › Code_and_Data/results/Figure2.pdf]

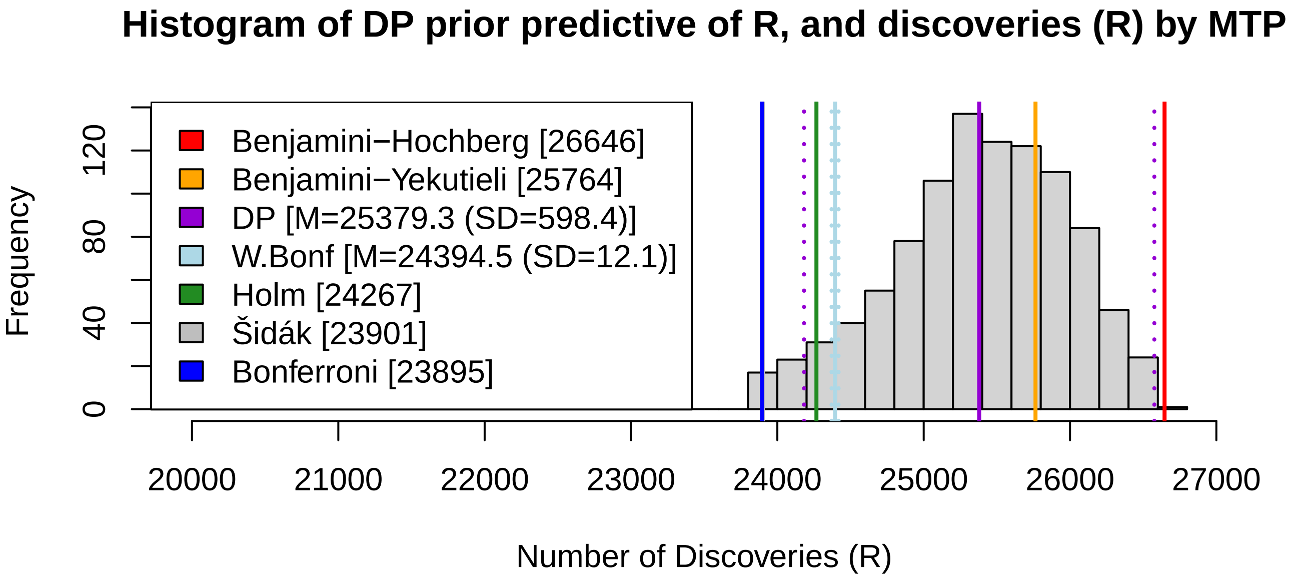

Histogram of DP prior predictive of R, and discoveries (R) by MTP
Benjamini−Hochberg [26646]
Benjamini−Yekutieli [25764]
DP [M=25379.3 (SD=598.4)]
W.Bonf [M=24394.5 (SD=12.1)]
Holm [24267]
Šidák [23901]
Bonferroni [23895]
Frequency
0
40
80
120
20000
21000
22000
23000
24000
25000
26000
27000
Number of Discoveries (R)

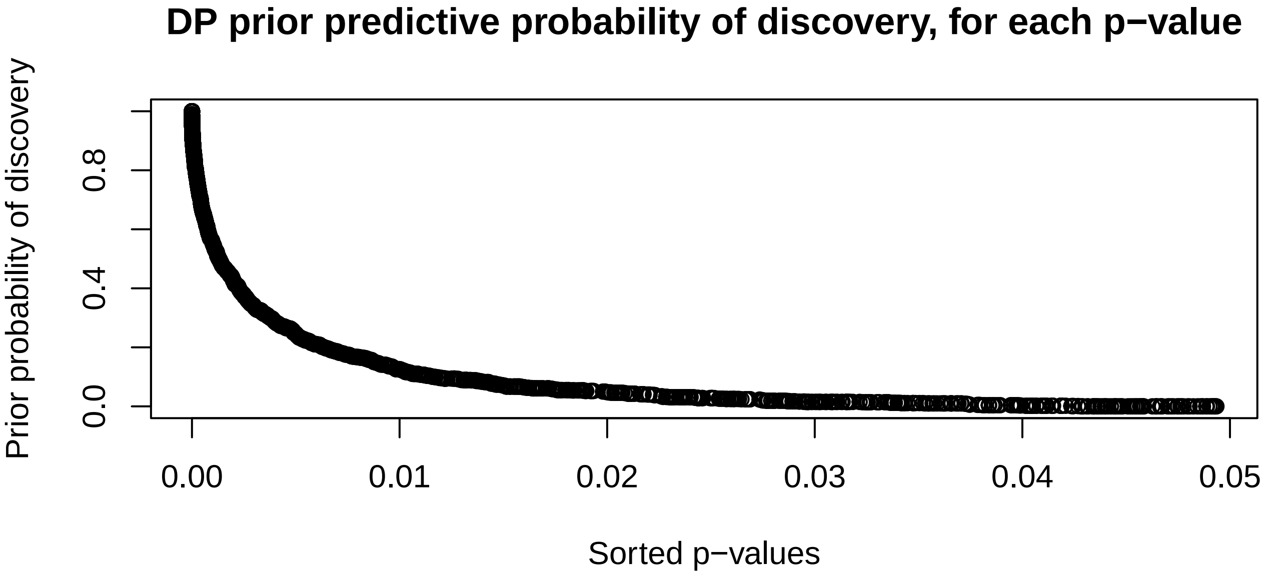

DP prior predictive probability of discovery, for each p−value
Prior probability of discovery
0.0
0.4
0.8
0.00
0.01
0.02
0.03
0.04
0.05
Sorted p−values
