# Supplementary figures and images for: Bayesian Nonparametric Sensitivity Analysis of Multiple Test Procedures Under Dependence
Source: Biom J. 2025 Dec 14;67(6):e70101. doi: 10.1002/bimj.70101 (PMC12703229; doi:10.1002/bimj.70101)

# Histogram of p-values, and number of discoveries (R) by MTP

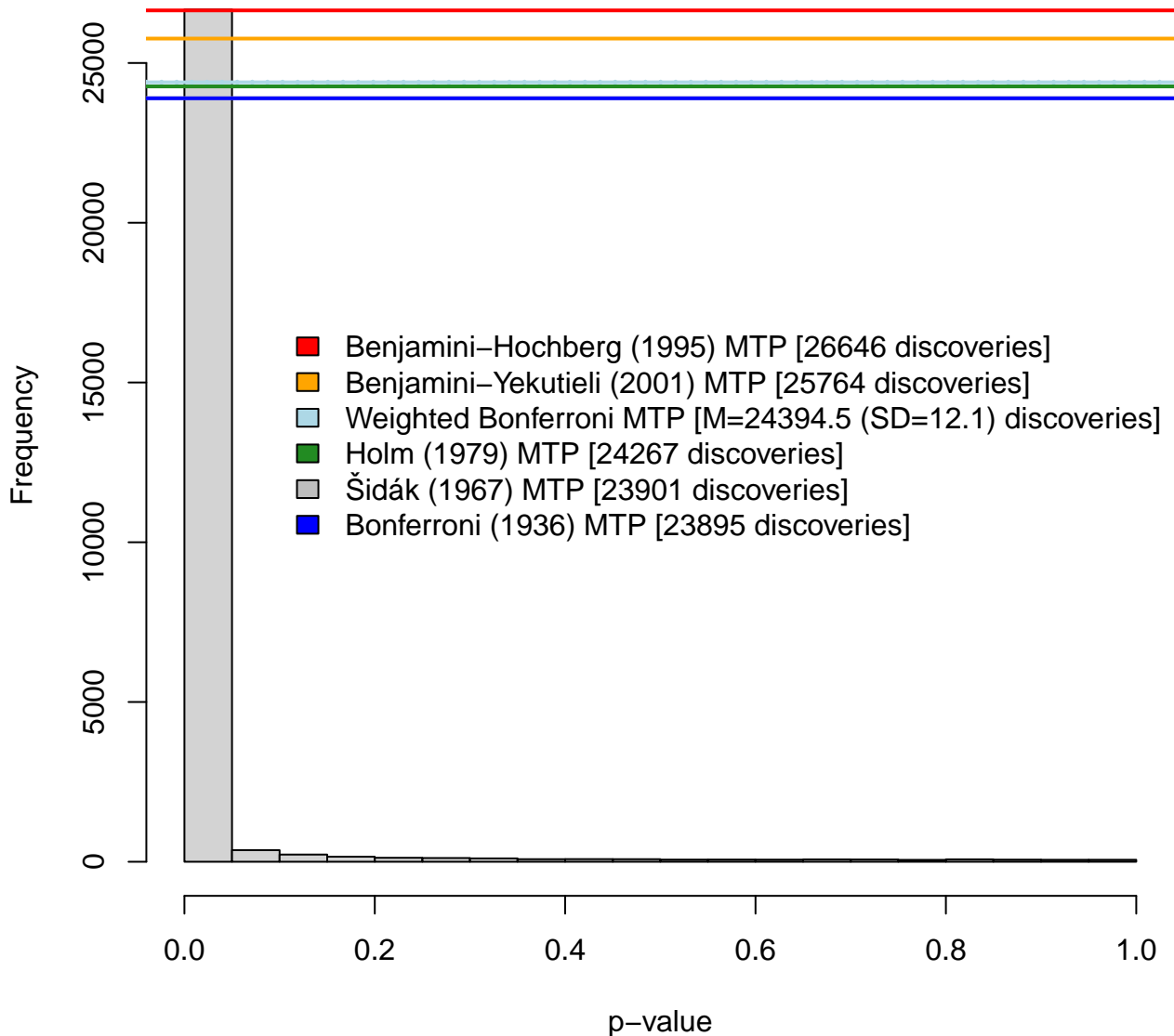

Supplement: Supplementary file 1 — Supporting File: bimj70101‐sup‐0001‐DataCode.zip [file BIMJ-67-e70101-s001.zip › Code_and_Data/results/Figure1.pdf]
